# An ancient conserved role for prion protein in learning and memory

**DOI:** 10.1242/bio.025734

**Published:** 2018-01-10

**Authors:** Patricia L. A. Leighton, Nathan J. Nadolski, Adam Morrill, Trevor J. Hamilton, W. Ted Allison

**Affiliations:** 1 Centre for Prions and Protein Folding Disease, University of Alberta, Edmonton, AB T6G 2M8, Canada; 2 Department of Biological Sciences, University of Alberta, Edmonton, AB T6G 2E9, Canada; 3 Department of Psychology, MacEwan University, Edmonton, AB T5J 4S2, Canada; 4 Neuroscience and Mental Health Institute, University of Alberta, Edmonton, AB T6G 2E1; 5 Department of Medical Genetics, University of Alberta, Edmonton, AB T6G 2H7, Canada

**Keywords:** Alzheimer, Anxiety, Learning, Novel object recognition, Targeted Mutagenesis, Zebrafish

## Abstract

The misfolding of cellular prion protein (PrP^C^) to form PrP Scrapie (PrP^Sc^) is an exemplar of toxic gain-of-function mechanisms inducing propagated protein misfolding and progressive devastating neurodegeneration. Despite this, PrP^C^ function in the brain is also reduced and subverted during prion disease progression; thus understanding the normal function of PrP^C^ in healthy brains is key. Disrupting PrP^C^ in mice has led to a myriad of controversial functions that sometimes map onto disease symptoms, including a proposed role in memory or learning. Intriguingly, PrP^C^ interaction with amyloid beta (Aβ) oligomers at synapses has also linked its function to Alzheimer's disease and dementia in recent years. We set out to test the involvement of PrP^C^ in memory using a disparate animal model, the zebrafish. Here we document an age-dependent memory decline in *prp2^−/−^* zebrafish, pointing to a conserved and ancient role of PrP^C^ in memory. Specifically, we found that aged (3-year-old) *prp2^−/−^* fish performed poorly in an object recognition task relative to age-matched *prp2^+/+^* fish or 1-year-old *prp2^−/−^* fish. Further, using a novel object approach (NOA) test, we found that aged (3-year-old) *prp2^−/−^* fish approached the novel object more than either age-matched *prp2^+/+^* fish or 1-year-old *prp2^−/−^* fish, but did not have decreased anxiety when we tested them in a novel tank diving test. Taken together, the results of the NOA and novel tank diving tests suggest an altered cognitive appraisal of the novel object in the 3-year-old *prp2^−/−^* fish. The learning paradigm established here enables a path forward to study PrP^C^ interactions of relevance to Alzheimer's disease and prion diseases, and to screen for candidate therapeutics for these diseases. The findings underpin a need to consider the relative contributions of loss- versus gain-of-function of PrP^C^ during Alzheimer's and prion diseases, and have implications upon the prospects of several promising therapeutic strategies.

## Abstract (precis)

**Summary:** Prion protein dysfunction at the synapse impacts learning in Alzheimer disease. Here, we demonstrate similar roles for prion protein in zebrafish, revealing ancient constructive roles for this infamously toxic protein.

## INTRODUCTION

Prion diseases are a unique class of neurological diseases that naturally affect a number of mammalian species including humans (e.g. Creutzfeld Jakob Disease, Fatal Familial Insomnia), cattle (Bovine Spongiform Encephalopathy; commonly known as mad cow disease), sheep (Scrapie), as well as deer and other cervids (Chronic Wasting Disease). The devastating impacts of these diseases span from the wellbeing of individuals to the socioeconomics of various industries and ecosystems. In these diseases, normal proteins (cellular prion protein, or PrP^C^) are converted to misfolded forms (prions), and the resulting prions propagate the diseases to neighbouring cells and tissues and infect new hosts. Despite identification of prions as disease agents in the early 1980s (Prusiner, 1982) and the creation of multiple lines of PrP^C^ knockout mice (Manson et al., 1994; Büeler et al., 1992; Sakaguchi et al., 1995; Yokoyama et al., 2001; Rossi et al., 2001; Moore et al., 1995), the normal functions of PrP^C^ remain ambiguous. PrP^C^ is a glycosylphosphatidylinositol (GPI)-anchored protein that is present within synapses (Sales et al., 1998; Stahl et al., 1987). It is highly expressed in several brain regions including the cortex, hippocampus, striatum and in the olfactory bulb to a lesser extent, suggesting that it plays a role in cognition (Sales et al., 1998). Some Creutzfeld-Jakob Disease patients have memory impairments (Caine et al., 2015), and PrP^C^ may contribute to cognitive decline in Alzheimer's disease (reviewed in Kostylev et al., 2015). Briefly, some forms of amyloid beta (Aβ) oligomers exhibit high-affinity binding to PrP^C^ (first reported in Lauren et al., 2009), ultimately leading to synaptic dysfunction (reviewed in Kostylev et al., 2015). In prion diseases and Alzheimer's disease, pathologies underlying memory impairments and other symptoms are thought to be mediated in part by PrP^C^ loss-of-function (for review see Leighton and Allison, 2016).

Several rodent behavioural studies have reported roles for PrP^C^ in memory and learning, though this has been controversial. Short-term social recognition memory was lower in the Zurich I line of *Prnp^−/−^* mice (ZrchI *Prnp^−/−^* mice) than in wild-type mice at 3 months of age, and prion protein (PrP) overexpression in Tg20 mice improved social recognition memory in 11-month-old mice relative to age-matched wild-type mice (Rial et al., 2009). Tg20 mice (transgenic line overexpressing *Prnp*) also had increased levels of synaptophysin compared to ZrchI *Prnp^−/−^* mice or wild-type mice (Rial et al., 2009), though it is unclear if this equates to a change in the number of synapses. ZrchI *Prnp^−/−^* mice exhibited reduced object recognition memory at 9 and 20 months of age compared to age-matched *Prnp^+/+^* mice, and both genotypes exhibited age-related memory impairments (Schmitz et al., 2014). Additionally, the Nagasaki line of *Prnp^−/−^* mice displayed an age-related decline in memory and/or latent learning in a water-finding test. This was not observed in age-matched *Prnp^+/+^* mice (Nishida et al., 1997). Further, multiple lines of *Prnp^−/−^* knockout mice show impairments in conditioned memory tasks, particularly in the 6- to 20-month age range (Criado et al., 2005; Nishida et al., 1997; Rial et al., 2009; Coitinho et al., 2003; Schmitz et al., 2014). In contrast, while 3-month-old ZrchI *Prnp^−/−^* mice performed comparably to age-matched *Prnp^+/+^* in a water maze spatial learning task, they exhibited a delay in learning when the platform position was changed (Büeler et al., 1992). Impaired spatial learning was more apparent in 5- to 6-month-old Edinburgh *Prnp^−/−^* mice using the Barnes Maze, and these impairments were rescued by transgenic expression of PrP^C^ in neurons (Criado et al., 2005). Fear conditioning tests have also produced mixed results in 3- to 6-month *Prnp^−/−^* mice (Nishida et al., 1997; Rial et al., 2009; Roesler et al., 1999; Schmitz et al., 2014; Coitinho et al., 2003), but there have been consistent reports of learning deficits in older (9- to 20-month-old mice) *Prnp^−/−^* mice compared to age-matched *Prnp^+/+^* mice (Rial et al., 2009; Coitinho et al., 2003; Schmitz et al., 2014). The finding that 9-month-old rats treated with α-PrP^C^ antibody exhibit deficits in fear-conditioned learning demonstrates that PrP^C^ has a role in learning in other closely related rodents (Coitinho et al., 2003).

There have been mixed reports in the field regarding whether PrP^C^ contributes to anxiogenic behaviour (Schmitz et al., 2014; Rial et al., 2009; Coitinho et al., 2003; Roesler et al., 1999). It has consistently been reported that 3-month-old ZrchI *Prnp^−/−^* mice do not behave differently to age-matched *Prnp*^+/+^ mice (Schmitz et al., 2014; Rial et al., 2009; Coitinho et al., 2003; Roesler et al., 1999); however, one study using older animals reported that *Prnp^−/−^* mice spent significantly more time in the open arms than *Prnp^+/+^* animals (Schmitz et al., 2014), while others found no difference between genotypes (Rial et al., 2009; Coitinho et al., 2003). Age-related reductions in anxiety were found in ZrchI *Prnp^−/−^* mice and *Prnp^+/+^* mice in two studies (Schmitz et al., 2014; Rial et al., 2009), but were not found in Tg20 mice, which overexpress PrP^C^ (Rial et al., 2009). In a third study, however, no age-related changes in anxiety were found in either ZrchI *Prnp^−/−^* mice or *Prnp^+/+^* mice, nor in rats treated with an α-PrP antibody (Coitinho et al., 2003).

**Fig. 1.**
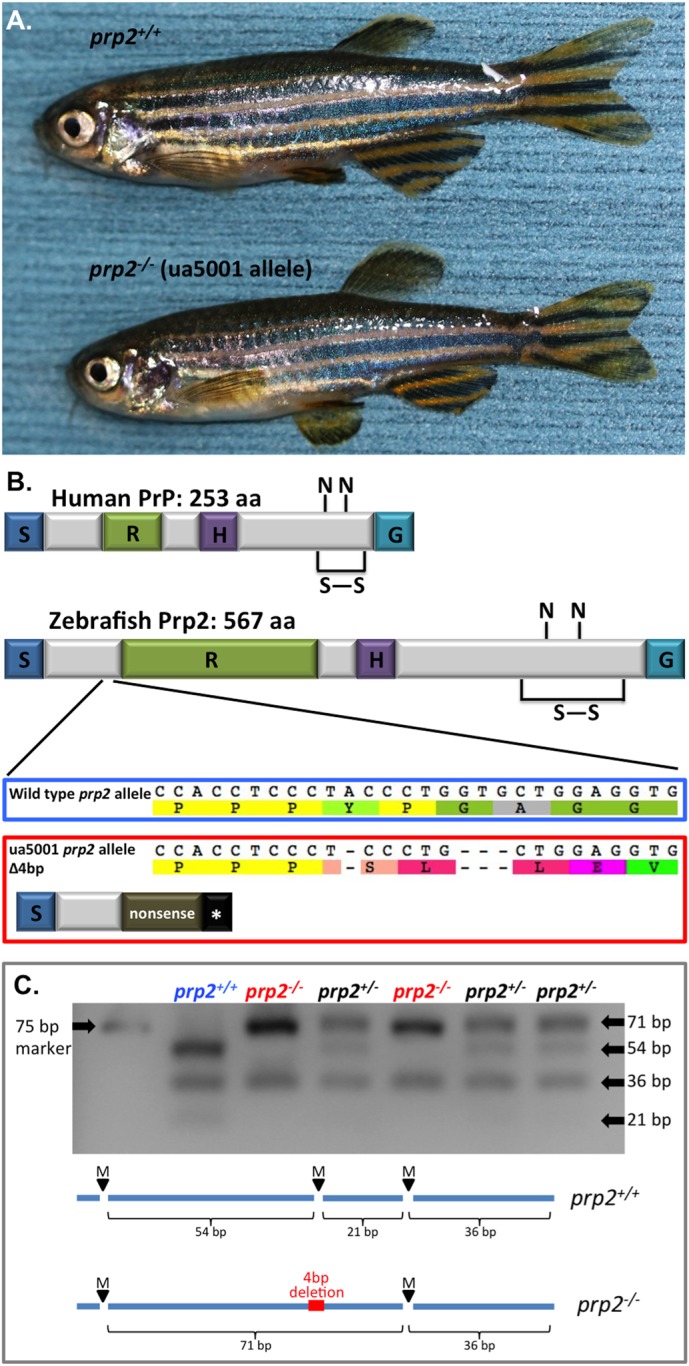
**Prion mutant *prp2^−/−^* zebrafish develop normally and display no overt phenotypes during adulthood.** (A) A young adult (∼1-year-old) *prp2^+/+^* fish is pictured on top, while a young adult *prp2^−/−^* fish is pictured on the bottom. (B) Zebrafish Prp2 is conserved with mammalian PrP^C^ at the protein domain level. Both have a signal peptide (S), a repeat region (R; though the repetitive region in zebrafish is longer and less patterned than the octarepeats in mammals), a hydrophobic domain (H) and are attached to the cell surface by a GPI anchor (G). Like mammalian PrP^C^, zebrafish Prp2 also has putative N-linked glycosylation sites (N) and a disulphide bond (S–S) within its C-terminus. The zebrafish *prp2* ua5001 allele has a 4 base pair deletion (frameshift), which produces an early stop codon and a putative nonsense protein lacking all these conserved domains. Further, the *prp2* gene product is greatly reduced in abundance, in *prp2^−/−^* mutant zebrafish, including in the brain tissue of adult fish (Fleisch et al., 2013). (C) Restriction fragment length polymorphism (RFLP) assay used for genotyping zebrafish at the *prp2* gene locus. There is an *Mva* cut site in the wild-type *prp2* sequence that is not present in the mutant (ua5001 allele) *prp2* sequence, leading to a smaller band when the PCR product from *prp2^+/+^* zebrafish is digested (54 bp) compared to from *prp2^−/−^* zebrafish (71 bp). bp, base pair.

An opportunity to reassess these proposed roles of PrP^C^ in memory and anxiety emerged from our recent engineering of *prp2^−/−^* zebrafish (Fleisch et al., 2013). These *prp2^−/−^* zebrafish are thought to be null mutants (or at least strong hypomorphs) because their frame shift mutation predicts loss of all recognizable domains from the mature protein (Fig. 1) and because the *prp2^−/−^* gene product is substantively and significantly decreased in abundance in these mutants, including within the adult brain tissues (Fleisch et al., 2013). Like *Prnp* knockout mice, *prp2^−/−^* zebrafish display no overt phenotypes in adulthood (Fig. 1). *Prp2^-/−^* zebrafish have altered N-methyl-D-aspartate (NMDA) receptor kinetics (Fleisch et al., 2013), and given that NMDA receptors play critical roles in learning and memory in various animals (reviewed in Morris, 2013), including in zebrafish (Swain et al., 2004), we predicted that fish lacking *prp2* would display memory impairments. Further, *prp2^−/−^* zebrafish have increased susceptibility to convulsants (Fleisch et al., 2013) and alterations in neural development (Huc-Brandt et al., 2014), encouraging the suggestion that synaptic function might be disrupted in a manner consistent with memory deficits. Zebrafish are an attractive model system for the study of disease because they reproduce in large numbers, can be deployed in high-throughput *in vivo* drug screens, have a sequenced annotated genome and are accessible for genetic manipulation (Norton and Bally-Cuif, 2010; Tierney, 2011). Regarding aging, zebrafish typically reach adulthood (sexual maturity) at about 3 months of age and display reduced fecundity after their second year, but often live to be 4 or 5 years old (Gerhard et al., 2002; Kishi et al., 2003). Although some important differences in brain structures exist between fish and mammals, the overall brain structure, cellular architectures and neurotransmitter systems are highly comparable between fish and mammals (Panula et al., 2010; Rodriguez et al., 2002; Norton and Bally-Cuif, 2010). A growing number of cognitive tests are being developed for use in zebrafish (Tierney, 2011), including those that assess both spatial and associative learning (reviewed in Norton and Bally-Cuif, 2010).

Object recognition memory has been used as a model of declarative memory (memory of facts, events and places) in rodents and zebrafish (Hammond et al., 2004; May et al., 2016). In rats it has been experimentally demonstrated that object recognition over short retention intervals involves the perirhinal cortex (Hannesson et al., 2004; Aggleton et al., 2010; Winters et al., 2011), while recognition over longer retention intervals requires the hippocampus (Hammond et al., 2004). The object recognition/preference test is a working memory test (Ennaceur and Delacour, 1988) that is commonly used in rodents (Hammond et al., 2004). Advantages of the object recognition test include its relative simplicity to perform (as it is a test of one-trial learning), and repetitive training with reinforcers are not required (Ennaceur and Delacour, 1988). Some of us recently established an object recognition test for adult zebrafish, and we found that wild-type zebrafish prefer the familiar object over the novel object, providing evidence for a functional object recognition memory system in zebrafish (May et al., 2016).

Methods to reliably test anxiety behaviour in zebrafish have also been introduced in recent years. Like rodents, zebrafish exhibit anxiety-like behaviour when exposed to novel environments. Novel tank diving tests and open-field tests are standard methods for measuring anxiety in zebrafish and have been evaluated pharmacologically (Maximino et al., 2010). The novel tank diving test exploits the innate tendency of several zebrafish strains to seek protection when exposed to novel environments (Egan et al., 2009). In this test, fish are typically placed in a narrower tank and bottom dwelling activity is used as the main output of anxiety (sometimes along with other measures such as erratic swimming, swimming bouts and thigmotaxis) (Maximino et al., 2010). In the open-field test, fish are placed in a novel (usually circular arena) and exploratory behaviour and thigmotaxis (wall hugging) are measured (Champagne et al., 2010; Maximino et al., 2010). The novel object approach (NOA) test (also known as the boldness test) is a variation of the open field test where an object is introduced into a circular arena after an acclimation period (Wright et al., 2003, 2006; Moretz et al., 2007; Johnson and Hamilton, 2017). Time spent near the object and away from the object (in the thigmotaxis zone) is then quantified. In a different test used to assess fear, computer simulated images of natural predators and select geometric shapes induced responses in domesticated zebrafish including freezing, erratic movement and more time spent on the side of the arena away from the stimulus (Ahmed et al., 2012). Thus avoidance of the novel object in the NOA test may be interpreted as an innate response to a perceived threat.

In this study we deployed our previously established object recognition/preference test (May et al., 2016) and found that zebrafish engineered to lack *prp2* show age-related declines in familiar object preference, suggesting their object recognition memory system is compromised. *Prp2^−/−^* fish did not display age-related differences in anxiety in the novel tank diving test. Using the NOA test, however, we found that 3-year-old *prp2^−/−^* fish approached the novel object more than the 1-year-old *prp2^−/−^* fish or age-matched *prp2^+/+^* fish, likely indicating an age-dependent change in cognitive appraisal of the object.

## RESULTS

### *prp2^−/−^* fish displayed an age-dependent decline in familiar object preference

**Fig. 2.**
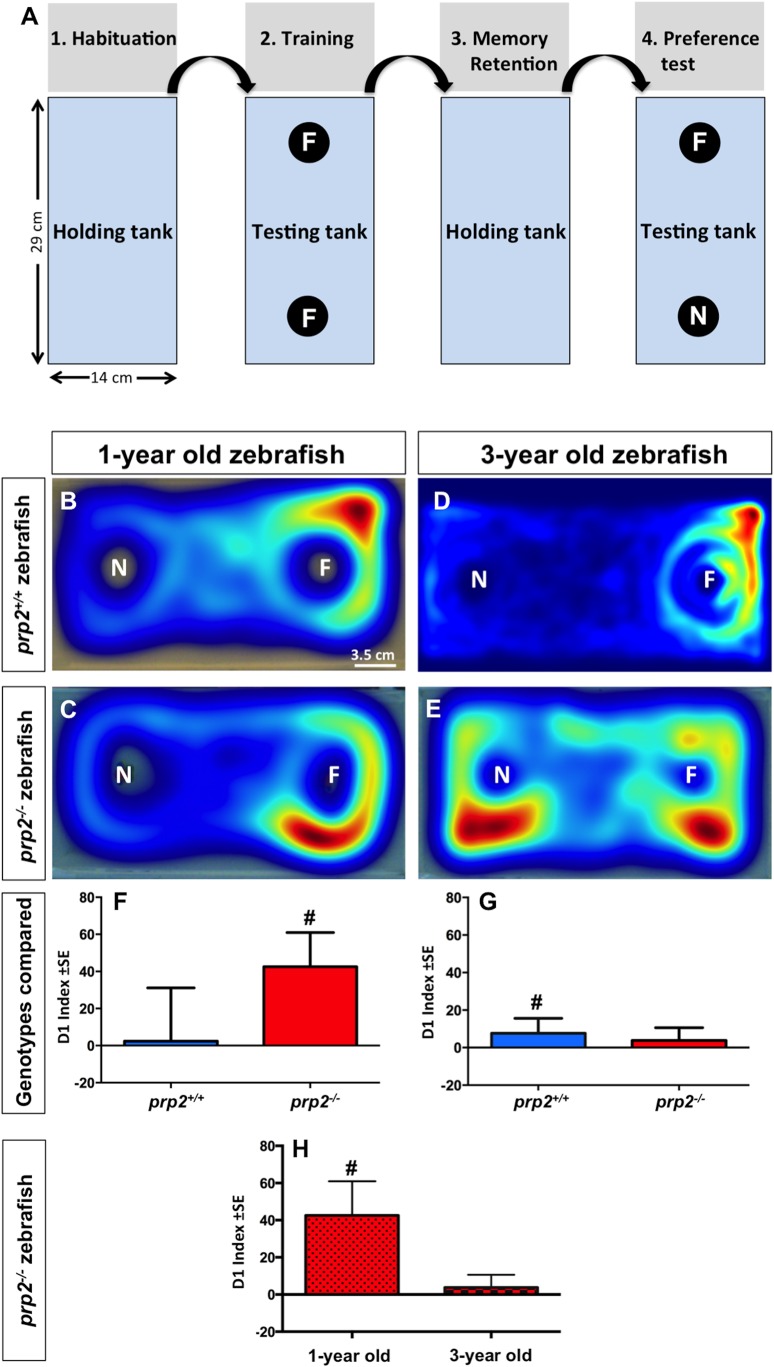
**Zebrafish lacking prion protein exhibited minor reductions in memory in an age-dependent fashion.** *Prp2^−/−^* fish showed a trend towards an age-dependent decline in familiar object preference with the object preference test. (A) Flowchart summarizing the sequence of events in the object preference test. (1) Fish were first habituated to a tank of the same size as the testing arena (the holding tank). (2) Fish were then netted and moved to the testing tank containing two identical objects (F) for the 10-min training phase. (3) Fish were then moved back to the holding tank for a 1- or 5-min period (memory retention interval), during which time one of the familiar objects (F) in the testing tank was replaced with a novel object (N). (4) Finally, fish were placed back into the testing tank for the 10-min object preference test. (B,C) Sample heat maps of 1-year-old *prp2^+/+^* fish and *prp2^−/−^* fish that displayed object preference during the test phase. Top down view of the test tank, wherein fish can swim around the novel object (N) and/or the familiar object (F). Warm colours (yellows and reds) in the heat map indicate this individual fish spent more time near the familiar object, which was interpreted herein as indicating the fish remembered this object from its earlier training phase (see Materials and Methods and assumptions in Discussion). Scale bar: 3.5 cm (the approximate size of an adult zebrafish). (D,E) Sample heat maps of 3-year-old *prp2^+/+^* fish and *prp2^−/−^* fish. This representative *prp2^+/+^* fish (D) exhibited familiar object preference during the test phase, while this example *prp2^−/−^* fish (E) did not (quantified across multiple replicates below). (F) 1-year-old *prp2^−/−^* zebrafish displayed familiar object preference following a 1-min retention interval, while the 1-year-old *prp2^+/+^* fish did not, as revealed by the D1 index of object preference (# indicates significant difference from 0 at *P*<0.05 using the Wilcoxon Signed Rank test; *n*=13 *prp2^+/+^* fish, *n*=28 *prp2^−/−^* fish). (G) 3-year-old *prp2^+/+^* fish displayed familiar object preference after a 1-min retention interval while 3-year old *prp2^−/−^* fish did not (D1 discrimination index, # indicates significant difference from 0 at *P*<0.05 using the Wilcoxon signed rank test; *n*=16 fish/genotype). (H) Zebrafish lacking prion protein (*prp2^−/−^*) displayed a notable, though not statistically significant, reduction in familiar object preference when comparing between ages as measured by D1 (values replotted from Fig. 2F,G).

Object preference tests were performed to assess memory in young (1-year-old) versus old (3-year-old) *prp2^−/−^* fish and to compare memory capacity (object preference) between *prp2^−/−^* and *prp2^+/+^* fish. The discrimination indices equations (D_1_, D_2_ and D_3_) are the most commonly used methods of quantifying object recognition in animal research. They take into account and compare the time the animal explores both objects and the total time that the animals are exploring either one during the testing phase. This compensates for differences in animals that either explore the objects a great deal or hardly at all. Researchers were blind to fish genotype during all behavioural testing. In these tests, the fish were first individually exposed to two identical objects on opposite sides of the tank (training phase, see Fig. 2A). The fish were then removed for a specified period of time representing the memory retention interval. Finally, the fish were tested in the same tank with an original (familiar) object on one side of the tank and a novel object on the other side; this represents the test phase. We quantified the amount of time each fish spent near each object during the test phase and calculated discrimination indices as described in Table 1. D1 is a discrimination index that measures the difference between time spent near the familiar object and the time spent near the novel object. The D2 and D3 discrimination indices account for the total time that the fish spend exploring the objects during the test phase. A1 and A2 are defined as the time spent near each of the two identical objects in Trial 1 (T1), a measure of exploration (E). In Trial 2 (T2), values A3 and B are defined as the time spent near the familiar object and the novel object, respectively.

**Table 1.**
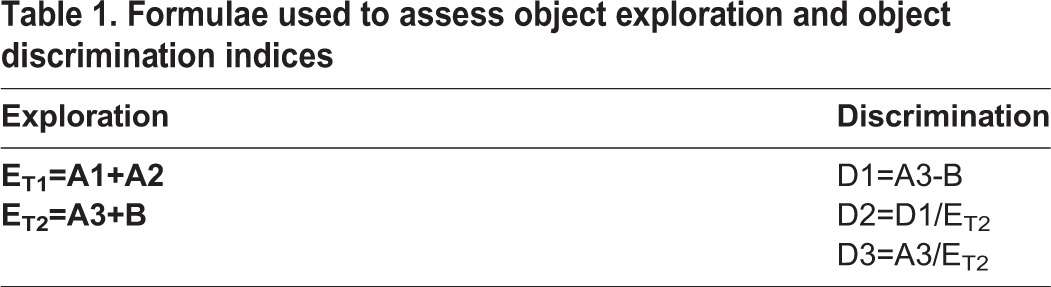
**Formulae used to assess object exploration and object discrimination indices**

**Fig. 3.**
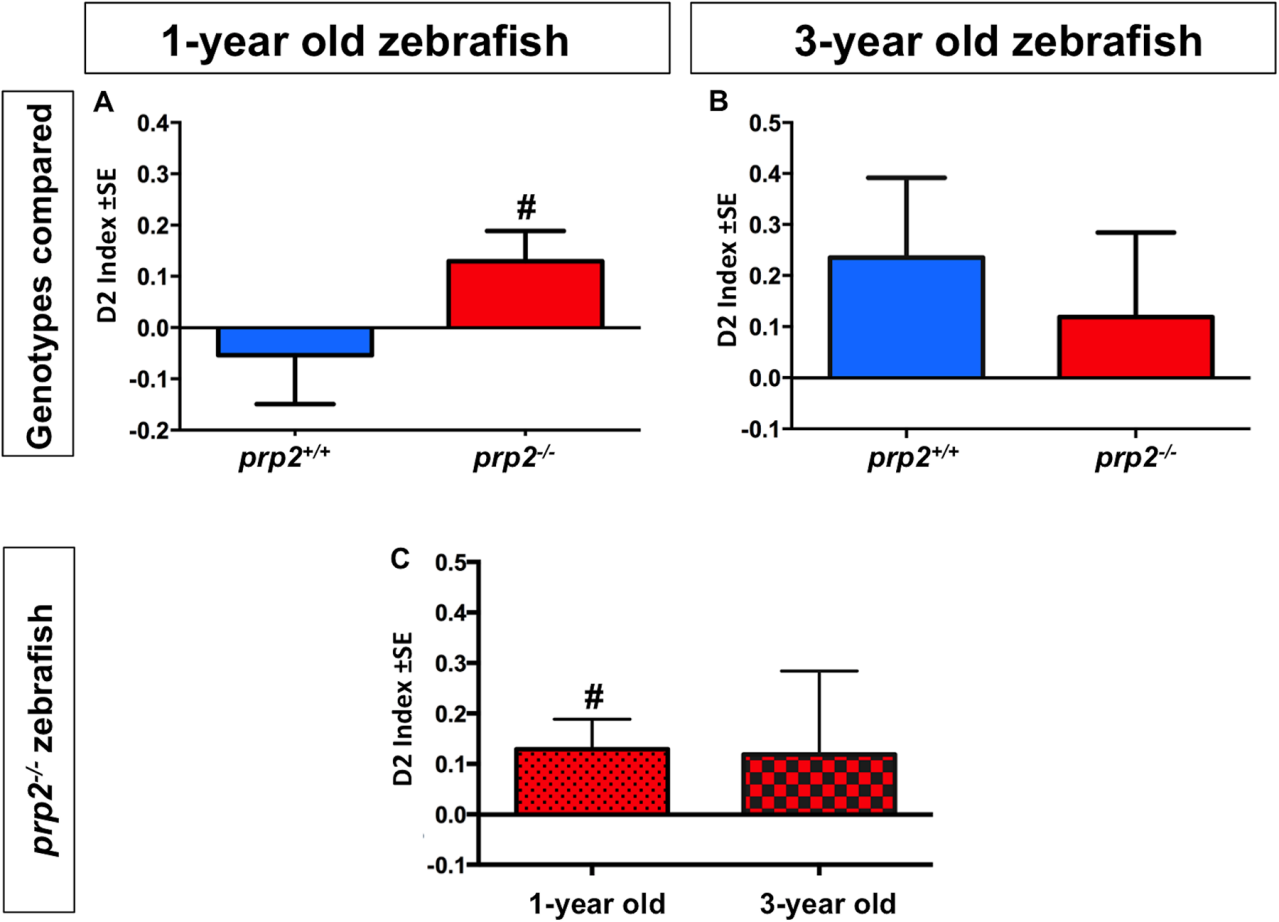
**Zebrafish lacking prion protein (*prp2^−/−^*) displayed an age-dependent decline in memory as revealed by the D2 discrimination index.** (A) 1-year-old *prp2^−/−^* zebrafish displayed familiar object preference following a 1-min retention interval, while the 1-year-old *prp2^+/+^* fish did not, as revealed by the D2 index of object preference (# indicates significant difference from 0 at *P*<0.05 using the one sample *t*-test; *n*=13 *prp2^+/+^* fish, *n*=28 *prp2^−/−^* fish). (B) 3-year-old fish of both genotypes (*prp2^+/+^* and *prp2^−/−^*) failed to show object preference following a 1-min retention interval using the D2 discrimination index (*n*=16 fish/genotype). (C) Zebrafish lacking prion protein (*prp2^−/−^*) displayed a small, though not statistically significant, reduction in familiar object preference with age as measured by D2 (values replotted from Fig. 3A,B).

These assays demonstrated that 1-year-old *prp2^−/−^* fish had learning and memory capabilities in a range typically observed in zebrafish, that is displaying object preference after a memory retention interval of at least 1 min (May et al., 2016) (Fig. 2). These 1-year-old *prp2^−/−^* fish had D1 and D2 discrimination indices >0 (Figs 2F and 3A; *P*<0.05) and D3 discrimination indices >0.5 after 1-min retention interval (Fig. S1A; *P*<0.05). This was in contrast to 3-year-old *prp2^−/−^* zebrafish, which did not display object preference after the 1-min retention interval (Figs 2G and 3B). This was not simply due to age, because 3-year-old wild-type *prp2^+/+^* fish displayed familiar object preference as measured by the D1 discrimination index (but not D2 or D3) (Figs 2G and 3B; Fig. S1B; *P*<0.05). Comparing the D1 and D2 discrimination indices of the 1-year-old *prp2^−/−^* fish to those of the 3-year-old *prp2^−/−^* fish, revealed a small (though not significantly different) trend towards reduced familiar object preference with age (Figs 2H and 3C, a re-plotting of the values in Figs 2F,G and 3A,B, respectively). Unexpectedly, 1-year-old *prp2^+/+^* fish did not display a significant object preference after a 1-min retention interval; however, this was likely due to the small sample size in this group (Figs 2F and 3A; Fig. S1A) (May et al., 2016).

### *prp2^−/−^* fish showed an age-dependent increase in approach to the novel object

A typical interpretation of the data in Figs 2 and 3 is that zebrafish lacking prion protein have reduced memory at old age. An alternative explanation for a lack of object preference among 3-year-old *prp2^−/−^* fish is that they perceive the objects differently compared to 3-year-old wild type fish and 1-year-old *prp2^−/−^* fish. In such an instance the novel objects might not invoke an innate anxious response or the zebrafish might not cognitively perceive the novel object as a threat. We addressed this hypothesis using the NOA test. In this test, zebrafish were first acclimated to a circular arena for 15 min and a novel object was then introduced into the centre of the arena for the last 5 min of the trial. The amount of time the fish spent in the object (centre) zone, middle zone, and thigomotaxis zone (outer edge of the arena) was calculated. Zebrafish spending less time in the thigmotaxis zone of the arena far from the object were interpreted to be less anxious (Johnson and Hamilton, 2017).

**Fig. 4.**
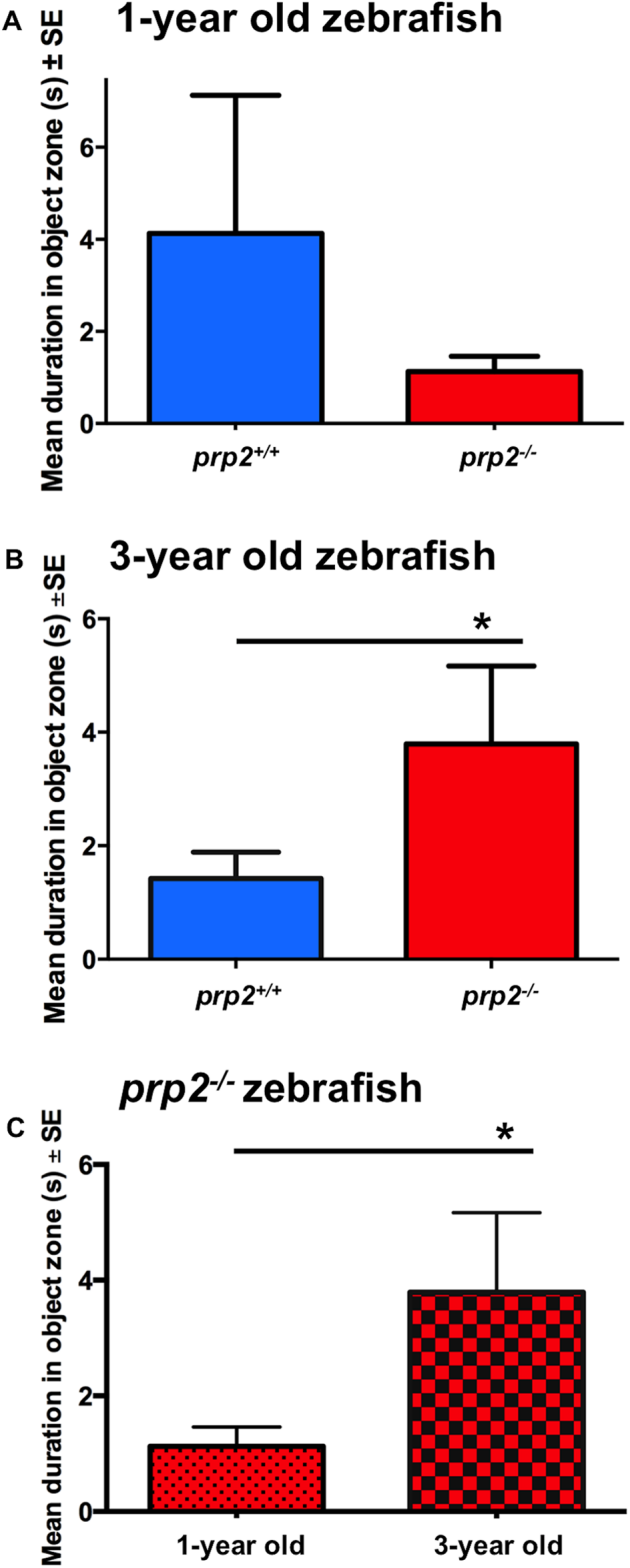
**Zebrafish lacking *prp2* exhibited an age-dependent difference in object appraisal.** 3-year-old *prp2^−/−^* fish spent more time in close proximity to the novel object than 1-year-old *prp2^−/−^* fish in the NOA test. (A) Amongst 1-year-old fish, there was no significant difference between genotypes (*prp2^+/+^* and *prp2^−/−^*) in time spent in the object (centre) zone (*n*=14 *prp2^+/+^* fish, *n*=29 *prp2^−/−^* fish). (B) Time spent in the object (centre) zone was significantly greater for the 3-year-old *prp2^−/−^* fish than for the 3-year- old *prp2^+/+^* fish (*P<0.05 with one-tailed Mann–Whitney test, *n*=16 fish/genotype). (C) 3-year-old *prp2 ^−/−^* fish spent a significantly greater period of time in the object (centre) zone than 1-year-old *prp2^−/−^* fish (*P<0.05 with the Mann–Whitney test; values replotted from Fig. 4A,B).

Among young (1-year-old) fish, there was no significant difference between genotypes in time spent in the object (centre) zone during the NOA test (Fig. 4A). Old (3-year-old) *prp2^−/−^* fish spent significantly more time in the object (centre) zone during the NOA test than 3-year-old *prp2^+/+^* fish (Fig. 4B; *P*<0.05). There were no differences in time spent in the middle zone or thigmotaxis zone between genotypes (data not shown). The 3-year-old fish also spent significantly more time in the object (centre) zone than the 1-year-old *prp2^−/−^* fish (Fig. 4C; *P*<0.05; a re-plotting of the values from Fig. 4A,B). Because no difference in time spent in the thigmotaxis zone was observed (an index of anxiety), but time spent in the centre (object) zone was significantly increased (an index of boldness of the object appraisal), this was suggestive of an age-dependent difference in object appraisal in the *prp2^−/−^* fish. Further assessments of anxiety were performed to assess this interpretation, below.

### No differences in anxiety were detectable between 3-year-old *prp2^+/+^* and *prp2^−/−^* fish genotypes, or with age in *prp2^−/−^* fish using the novel tank diving test

**Fig. 5.**
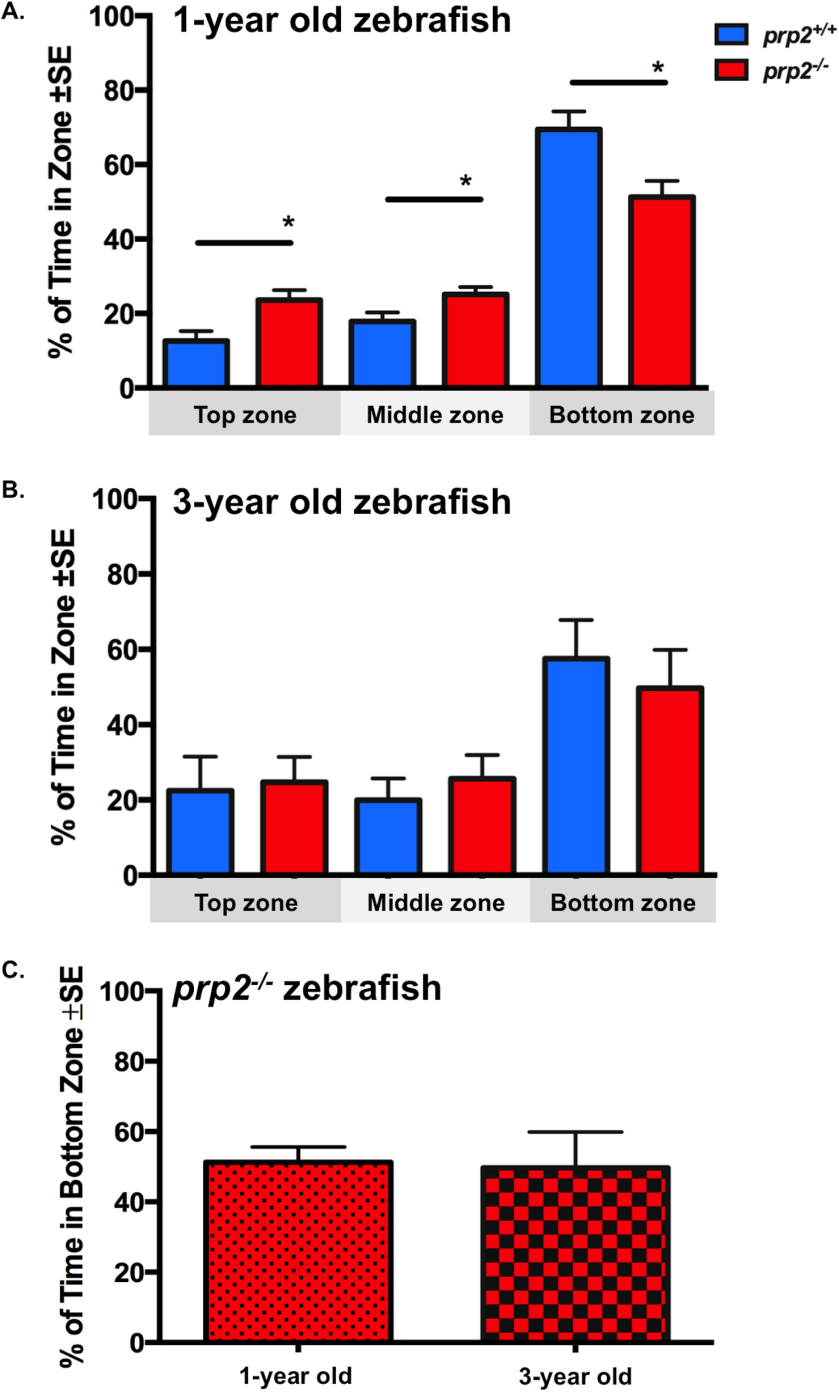
**There were no detectable differences in anxiety between 3-year-old *prp2^+/+^* and *prp2^−/−^* fish or age-related changes in anxiety among *prp2^−/−^* fish using the novel tank diving test.** All groups of fish displayed the typical bottom dwelling response to tank novelty. (A) The 1-year-old *prp2^+/+^* fish spent more time in the bottom zone and less time in the top and middle zones than age-matched *prp2^−/−^* fish (*P<0.05 with the unpaired *t*-test; *n*=14 *prp2^+/+^* fish, *n*=29 *prp2^−/−^* fish). (B) Among 3-year-old fish, there were no significant differences between genotypes in time spent in the top zone, middle zone, or bottom zone with the unpaired *t*-test (*n*=11 *prp2^+/+^* fish, *n*=10 *prp2^−/−^* fish). (C) 1-year-old *prp2^−/−^* fish and 3-year old *prp2^−/−^* fish spent a comparable proportion of time in the bottom zone (values replotted from Fig 5A,B).

The novel tank diving test, an established and sensitive anxiety test (Maximino et al., 2010), was deployed to determine differences in anxiety. Such differences might have accounted for reduced object preference and increased NOA observed with age or between genotypes. The zebrafish were exposed to a tank that was narrower and deeper than their home tank; the time the fish spent in the bottom, middle and top third of the tank was recorded. In this test, ‘bottom dwelling’ is considered an anxious response. Consistent with previous reports (Bencan et al., 2009), our wild-type (*prp2^+/+^*) fish of both ages exhibited an anxious response to the novel environment: they spent proportionally more time in the bottom zone than in the top zone of the novel tank (Fig. 5A,B). The 1-year-old *prp2^+/+^* fish spent significantly more time in the bottom zone, and significantly less time in the top and middle zones than the 1-year-old *prp2^−/−^* fish in the novel tank diving test (Fig. 5A; *P*<0.05), indicating that the *prp2^+/+^* fish were more anxious. This increase in anxiety among 1-year-old *prp2^+/+^* fish might contribute to their unexpected lack of object preference. There were no significant differences between aged (3-year-old) fish of the *prp2^−/−^* and *prp2^+/+^* genotypes in the top zone, middle zone or bottom zone of the tank during the novel tank diving test (Fig. 5B). Further, there were no age-dependent differences in the time the *prp2^−/−^* fish spent in the bottom zone (Fig. 5C), suggesting that these fish displayed no age-dependent changes in anxiety.

## DISCUSSION

The goal of our study was to determine whether PrP^C^ has a conserved role underlying memory and anxious behaviour. We also sought to characterize a zebrafish PrP^C^ loss-of-function model that could be used for testing potential prion disease and Alzheimer's disease therapeutics in the future. There are many advantages of using zebrafish as a model for drug testing that include (1) water soluble drugs can be applied directly to the tank water and thus drug delivery is not invasive; and (2) drugs can be applied continuously, aiding study of drug pharmacokinetics (Kedikian et al., 2013).

### PrP^C^ influences object preference in zebrafish, a role that is conserved in mice

We used a zebrafish object preference paradigm (May et al., 2016) to assess object recognition memory in our recently engineered *prp2^−/−^* fish (Fleisch et al., 2013). Similar to rodent novel object preference paradigms (Dodart et al., 1997; Ennaceur and Delacour, 1988), we analysed the time the fish spent exploring (i.e. in close proximity to) a novel object compared to time spent exploring a familiar object. We used previously established discrimination indices (Table 1) (May et al., 2016; Akkerman et al., 2012) to assess novel object preference in young (1-year-old) and aged (3-year-old) *prp2^−/−^* fish. Using the D1, D2, and D3 discrimination indices, we found that 1-year-old *prp2*^−/−^ fish displayed preference for the familiar object, similar to what was previously found for wild-type fish. We interpret this familiar object preference as a response to recognizing the familiar object. Using the D1 discrimination index, we found that 3-year-old *prp2*^+/+^ zebrafish displayed familiar object preference after a 1-min retention interval, while 3-year-old *prp2^−/−^* fish did not. When taking exploration time into account using the D2 and D3 discrimination indices, however, the 3-year-old *prp2*^+/+^ fish also did not display familiar object preference. When we compared the D1 and D2 indices of the 1-year-old *prp2^−/−^* fish to those of the 3-year-old *prp2^−/−^* fish, we found reduced object preference among the older fish (though this did not reach statistical significance, perhaps due to the small sample size), which suggests that *prp2*^−/−^ fish exhibit age-dependent memory decline. This age-dependent decline in object discrimination is comparable to what has been reported in *Prnp^−/−^* mice using a novel object recognition paradigm (Schmitz et al., 2014).

### PrP influences object recognition and cognitive appraisal in zebrafish

While object preference has been previously used as a proxy for object recognition, alternative explanations for the age-dependent decline in object preference among *prp2^−/−^* fish include changes in cognitive appraisal and/or anxiety. In the NOA test the aged (3-year old) *prp2^−/−^* fish spent more time exploring the novel object than the 3-year-old *prp2^+/+^* fish and the young (1-year-old) *prp2^−/−^* fish. In this test, fish that keep distance from the object and spend time in the thigmotaxis zone could be interpreted as exhibiting fear of a predator (Maximino et al., 2010), and it is possible that the fish fear the object due to its relative size (May et al., 2016). If this were the case, the older *prp2^−/−^* fish would be interpreted as having adopted a more risky/bold behaviour, or they may not appraise the object as being one to fear. It was previously found that *Prnp^−/−^* mice exhibited less anxiety in an elevated plus maze than *Prnp^+/+^* mice following acute stress (foot shock or swimming in a tank of water) (Nico et al., 2005). This may mean that PrP^C^ is involved in adapting to conditions of stress (Nico et al., 2005). However, the interpretation that the 3-year-old *prp2^−/−^* fish have decreased anxiety is not consistent with findings showing that the anxiolytic drug ethanol reduces time spent in the thigmotaxis zone, but does not change time spent near the object (Johnson and Hamilton, 2017). We observed no change in time spent in the thigmotaxis zone in the 3-year-old *prp2^−/−^* fish suggesting no change in anxiety relative to 1-year-old *prp2^−/−^* fish or age matched controls (note, though that this argument is building towards the conclusion that the mutant fish are not more anxious; other types of anxiety may exist and we cannot prove this negative). A lack of change in anxiety-like behaviour in 3-year-old *prp2^−/−^* fish is consistent with our results from the novel tank diving test. In this test we saw no significant differences in bottom dwelling time (a proxy for increased anxiety) or top dwelling time (a proxy for decreased anxiety) in the 3-year-old *prp2^−/−^* fish compared to age matched *prp2^+/+^* fish or 1-year-old *prp2^−/−^* fish. The novel tank diving test is considered to be a more sensitive anxiety test compared to the novel approach test so it is unlikely that the *prp2^−/−^* fish are less anxious. An alternative explanation is that the 3-year-old *prp2^−/−^* fish have lost their ability to cognitively discern whether the novel object appears to be a predator, and such explanations might include changes to sensory systems such as visual system deficits. In other words, they may not recognize that the object is something to be afraid of. Indeed we cannot rule out that the prion mutant fish are generally aging faster than wild types, and various changes in physiology could manifest in changes in behaviour. Because we did not observe overt differences in behaviour, and because the indices we calculate normalize for changes in activity levels, the most obvious interpretation is that loss of prion protein has led to modest reductions in learning and memory.

Interestingly, the 1-year-old wild-type fish used in our study showed a non-significant trend towards a familiar object preference consistent with the strong preference observed in young wild-type fish used in a previous study (May et al., 2016). The lack of significant preference observed here is likely due to the small sample size of this group (*n*=13) compared to previous research (*n*=51) (May et al., 2016). Regardless, our most valid comparison is between the 1-year-old and 3-year-old *prp2^−/−^* fish, which demonstrated a loss of object recognition memory and cognitive appraisal.

In summary, we interpret our results as supporting the hypothesis that prion protein of zebrafish is required for learning and memory functions, and ruled out alternative explanations for the data that invoke differences in anxiety levels between genotypes. This is similar to the effects of *Prnp* loss on novel object recognition demonstrated previously in mice, supporting a conserved, ancient (and thus presumably important) role for prion protein in learning and memory.

### Potential cellular mechanisms linking PrP^C^ to memory and cognitive appraisal

PrP^C^ is a known interaction partner of many other membrane proteins and may contribute to memory formation through multiple mechanisms. PrP^C^ interactions with Sti1 and Laminin-γ1 have been shown to be involved in a memory paradigm in rats (Coitinho et al., 2006, 2007), and these interactions activate PKA and ERK 1/2 signalling (Coitinho et al., 2006; Beraldo et al., 2010). The PrP^C^-Sti1 complex also interacts with the α7 nicotinic acetylcholine receptor (Beraldo et al., 2010), which is a known regulator of long-term memory (reviewed in Jeong and Park, 2015). Low doses of nicotine enhance spatial recognition in zebrafish and antagonists of several zebrafish nicotinic acetylcholine receptors are available (Braida et al., 2014). Thus it would be possible to treat zebrafish *prp2^−/−^* fish with nicotine and nicotinic acetylcholine receptor antagonists to determine whether interactions between PrP^C^ and nicotinic acetylcholine receptors are important for memory retention. NMDA receptors have also been shown to be involved in zebrafish memory (Swain et al., 2004), and given that PrP^C^ regulates NMDA receptors, including in zebrafish (Khosravani et al., 2008; Stys et al., 2012; Fleisch et al., 2013), it would be interesting to investigate the effect of this regulation on object recognition memory.

A potential explanation for reduced cognitive appraisal in older *prp2^−/−^* fish could be reduced activity of nitric oxide synthase. Both Scrapie-infected mice and *Prnp^−/−^* mice exhibit alterations in the localization and activity of nitric oxide synthase (Keshet et al., 1999), and inhibition of nitric oxide synthase has been shown to increase exploratory behaviour of mice in an elevated plus maze, including time spent in the open arms and number of entries into the open arms (Volke et al., 1995). This altered behaviour may also be due to loss of regulation of nicotinic acetylcholine receptors by PrP^C^. Low doses of nicotine enhance cognitive functions, including memory, in zebrafish and mammals (reviewed in (Levin et al., 2006)). Thus if PrP^C^ interaction with nicotinic acetylcholine receptors (Beraldo et al., 2010) enhances memory, nicotinic acetylcholine receptor agonists may counteract memory deficits in aged *prp2^−/−^* fish. In turn, nicotine would be predicted to have a greater effect in *prp2^+/+^* fish than in *prp2^−/−^* fish.

### Conclusions and future outlook

Here we have demonstrated that zebrafish have object recognition memory and that this memory is disrupted by targeted mutagenesis of one of the zebrafish *Prnp* paralogs. We have recently engineered compound *prp1^−/−^*; *prp2^−/−^* zebrafish and when they have aged it will be important to determine whether loss of *prp1* exacerbates the age-dependent deficits in memory that we observed in our *prp2^−/−^* fish. Our zebrafish paradigm is relatively simple and well suited for testing which PrP^C^ interacting partners are important for mediating memory and synaptic plasticity *in vivo*, since drugs (e.g. nicotine, nicotinic receptor antagonists, MK-801) can be delivered by adding them to the tank water. Knowledge gathered from the object recognition memory paradigm will be applied to conditional learning paradigms to assess the roles of PrP^C^ and its interaction partners in learning. One such interaction partner is amyloid precursor protein (APP), and we have previously shown that zebrafish paralogs of APP and PrP^C^ interact during zebrafish development (Kaiser et al., 2012). As PrP^C^ is associated with prion diseases as well as Alzheimer's disease (through its interactions with APP and Aβ oligomers), knowledge gained from these studies will accelerate/enhance the development and screening of prion disease and Alzheimer's disease therapeutics.

Further, our data strongly support the growing list of phenotypes observed in prion loss-of-function models that map with high fidelity onto prion disease symptomology (Leighton and Allison, 2016; Allison et al., 2017). Thus, in contradistinction to the simplifying assumption that protein gain-of-function is largely responsible for disease outcomes, we infer that the aetiology of prion diseases likely requires prion protein function to be at least partially lost or subverted on the path to dementia.

## MATERIALS AND METHODS

### Zebrafish strains and husbandry

Zebrafish of the AB strain were used as the wild-type fish in this study. The *prp2^ua5001/ua5001^* zebrafish mutants (ZFIN ID: ZDB-ALT-130724-2) that we previously engineered (Fleisch et al., 2013), denoted as *prp2^−/−^* throughout this text, were generated and maintained on an AB strain background. *prp2^−/−^* zebrafish are thought to be null mutants, engineered by targeted mutagenesis to have a 4 base pair deletion in the beginning of the *prp2* coding region (which is contained within a single exon) leading to a protein that is predicted to be truncated and lack all recognizable prion protein domains; (Fleisch et al., 2013) (Fig. 1). In these mutants the *prp2* gene product is greatly reduced in abundance presumably by nonsense-mediated decay, including in adult brain tissue (Fleisch et al., 2013). *prp2^−/−^* fish used in the current study were maternal zygotic mutants at the *prp2* gene locus, but previous generations of fish were genotyped using a newly developed restriction fragment length polymorphism (RFLP) assay as described below (Fig. 1C). Wild-type zebrafish, denoted *prp2^+/+^* (AB background fish from the same stock as *prp2^−/−^* fish, such that mutants and wild types were closely related), were tested for comparison. The mean lifespan of laboratory raised zebrafish is ∼40 months (3.3 years) (Gilbert et al., 2014; Gerhard et al., 2002). In the current study, both young adult zebrafish (1 year old) and aged zebrafish (3 years old) were used. Fish of both ages displayed normal health and movement. The fish were raised and maintained within the University of Alberta fish facility at 28°C under a 14/10 light/dark cycle as previously described (Fleisch et al., 2013). Fish were transferred across town (4 kilometres) to the MacEwan University fish facility at least 2 weeks prior to the initiation of behavioural tests, where they were maintained as described in May et al. (2016). The MacEwan researchers performing the behavioural tests were blind to the genotype of the fish. Fish were transferred to MacEwan and tested in three separate batches separated by several months: the first being the 3-year-old *prp2^−/−^* and *prp^+/+^* fish, followed by the 1-year-old *prp2^−/−^* fish (denoted ‘ZF1’) and *prp^+/+^* fish (ZF2), then an additional group of 1-year-old *prp^+/+^* fish (ZF3) to increase the sample size for the 1-year-old control group. Prior to combining the control groups ZF2 and ZF3, we tested for significant differences and found a difference in velocity in T1 between groups ZF2 (5.5±0.2 cm/s, *n*=11) and ZF3 (7.9±0.4 cm/s, *n*=15) (*P*<0.01) suggestive of an altered behavioural state in ZF3 so this group was removed from the study. Exclusion of the ZF3 group further meant that all mutant and wild-type fish within each age group were treated in the most identical manner feasible with respect to time of transport and husbandry conditions.

All protocols were approved by the University of Alberta's Animal Care and Use Committee: Biosciences and the MacEwan University Animal Research Ethics Board (AREB), in compliance with the Canadian Council on Animal Care (CCAC).

### Genotyping

An RFLP assay was developed to genotype zebrafish at the *prp2* gene locus wherein the ua5001 mutation disrupted an *MvaI* cut site. Genomic DNA was amplified using *prp2* RFLP primers (forward primer 5’-TCC CCT GGA AAC TAT CCT CGC CAA C-3’; reverse primer 5’-TGG GTT AGA GCC TGC TGG TGG-3′), and then digested with Fast Digest *MvaI* (Thermo Fisher Scientific). PCR products from mutant and wild type DNA produced different banding patterns following electrophoretic separation (*prp2* wild-type allele yields three bands with sizes of 21, 36 and 54 base pairs; *prp2^−/−^* ua5001 allele yields two bands with sizes of 36 and 71 base pairs; Fig. 1C).

### Object preference/recognition test

The object preference/recognition test is designed to measure object recognition memory, and was structured to be a minor variant on the ‘novel object recognition’ (NOR) test that is prevalent in rodent research. The method exploits the observation that zebrafish presented with a novel and a familiar object spent more time near the familiar object relative to the novel object. Thus, similar to rodent research where innate preferences of novel objects are exploited to test memory, in our method the time zebrafish spent amongst novel and familiar objects is interpreted as familiar object preference and is considered a proxy for object recognition (i.e. memory) (May et al., 2016). The object preference test was performed between the hours of 09:00-17:00 as previously described (May et al., 2016). Briefly, fish were first placed in a holding tank for 5 min to acclimate. Fish were then netted and moved to a new tank that was identical to the holding tank, except for including the presence two identical objects for the zebrafish to explore (all objects devised from LEGO® pieces; see May et al., 2016) for a 10-min training trial (T1). Next, fish were moved back to the holding tank for either a 1-min or 5-min retention interval (RI). During this time an identical object in the trial tank was replaced with a novel object. The objects were randomly counterbalanced such that the object designated as familiar versus novel was randomized amongst fish. Finally, fish were moved back into the trial tank for a 10-min testing trial (Fig. 2A). Position and movement of zebrafish was recorded by an overhead camera and tracked in Ethovision XT (version 10.0, Noldus, VA, USA). To quantify the object preference for each fish we used the discrimination indices D1, D2 and D3 (Table 1) for the time fish spent in close proximity to the objects (8.4 cm^2^ boxes were placed over the objects in Ethovision) (May et al., 2016). Positive values of D1 and D2 that were significantly different from zero were interpreted to indicate a familiar object preference (negative values indicate a novel object preference). Values of D3 that were significantly different from 0.5 were also interpreted to indicate an object preference (greater than 0.5 indicates a familiar object preference whereas a value less than 0.5 indicates a novel object preference).

### NOA test

The NOA test is a two-phase test designed to measure the anxiety levels in a zebrafish exposed to a novel object. In the first phase of this test, the zebrafish were introduced using a small net into a circular arena (34 cm in diameter) filled with habitat water maintained between 26-28°C to a height of 5 cm. The trial was recorded using an overhead camera and tracked using Ethovision XT motion tracking software. This allowed for quantification of locomotion and thigmotaxis (wall hugging). After the first 15 min, phase two was initiated by the introduction of a novel object (as above, Ou et al., 2015) in the centre of the arena. The zebrafish was then recorded for an additional 5 min before terminating the trial. The circular arena was divided into three radial zones: the outer thigmotaxis zone, the middle (transition) zone, and the centre (object) zone (Fig. S2A). Increased anxiety is inferred from fish spending more time in the outer thigmotaxis zone and decreased boldness is inferred from fish spending less time near the object.

### Novel tank diving test

Anxiety levels of the zebrafish were also assessed using the novel tank diving test (Egan et al., 2009; Bencan et al., 2009; Parker et al., 2014). In this test zebrafish were netted and transferred into a tall, narrow, but deep rectangular arena measuring 24.9 cm×4.8 cm×18.1 cm, with glass walls 0.7 cm thick. The arena was filled with habitat water maintained between 26-28°C. We chose to use a rectangular rather than trapezoidal arena used in other studies (Egan et al., 2009; Parker et al., 2014) because we housed zebrafish in a trapezoidal tank [Aquatic Habitats (AHAB), Aquatic Ecosystems, Inc. Apopka, FL, USA] so our choice or a ‘novel tank’ for the diving test would be relatively more novel than a thinner trapezoidal tank. The location of the fish was recorded, using a camera positioned at the side of the tank, and analyzed with Ethovision XT motion tracking software for 5-min trials. The arena was divided into three equal latitudinal zones; the Top Zone, Middle Zone, and Bottom Zone (Fig. S2B). Zebrafish that spend more time in the bottom of the arena, similar to rodents spending more time in the closed arms of an elevated plus maze or near the walls of an open field arena, were considered to have elevated anxiety relative to fish that explored the upper areas of the arena.

### Statistics

Data were analyzed using GraphPad Prism Software (San Diego, CA, USA). For one sample testing, normality was first assessed using D'Agostino and Pearson omnibus normality tests. Parametric data were analyzed using one sample *t*-tests, and nonparametric data were analyzed using Wilcoxon signed rank tests. For multiple sample comparisons, variances were first assessed using F-tests. Parametric data were then analyzed with unpaired *t*-tests, and nonparametric data were analyzed with Mann–Whitney tests. Well-established discrimination indices typical of object recognition tests (D1, D2 and D3) were used to assess object preference as described previously (Table 1) (May et al., 2016).

## Supplementary Material

Supplementary information
