# Supplementary material for: An ancient conserved role for prion protein in learning and memory
Source: Biol Open. 2018 Jan 10;7(1):bio025734. doi: 10.1242/bio.025734 (PMC5829491; doi:10.1242/bio.025734)
Supplement: Supplementary information [file biolopen-7-025734-s1.pdf]

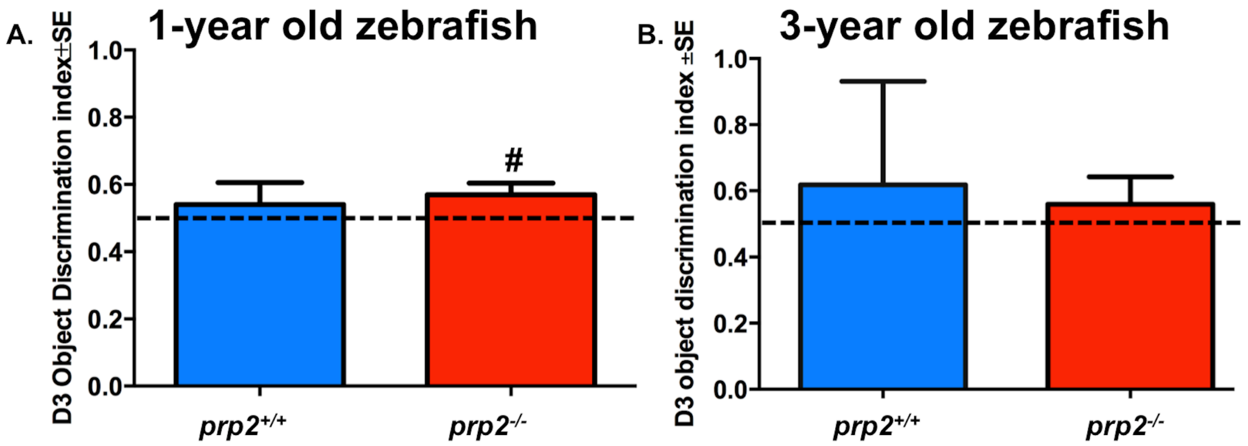

Supplementary Figure S1. After a 1-minute retention interval, 1-year old *prp2*$^{-/-}$ zebrafish displayed familiar object preference with the object preference test, but 3-year old *prp2*$^{-/-}$ zebrafish did not. **A.** The D3 discrimination index revealed that 1-year old *prp2*$^{-/-}$ zebrafish displayed familiar object preference after a 1-minute retention interval, but 1-year old *prp2*$^{+/+}$ zebrafish did not. # indicates significant difference from 0.5 (dotted line) with $p<0.05$ using the one-sample t-test; n=13 *prp2*$^{+/+}$ fish, n=28 *prp2*$^{-/-}$ fish). **B.** At 3 years of age neither genotype (*prp2*$^{+/+}$ or *prp2*$^{-/-}$) displayed object preference after a 1-minute retention interval as revealed by the D3 discrimination index (n=16 fish/genotype).

Biology Open • Supplementary information

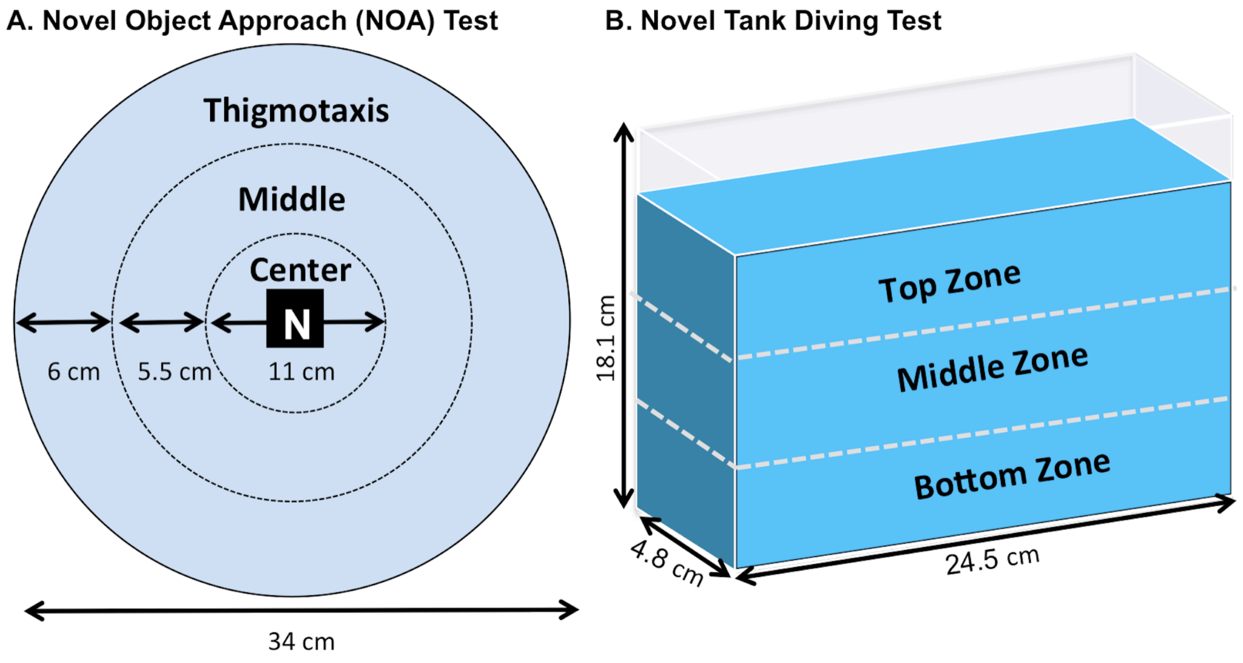

Supplementary Figure S2. A. Schematic of the Novel Object Approach (NOA) setup. Fish were netted and placed into a circular arena, where they acclimated for 15 minutes. After this time, a novel object (N) was placed into the center of the arena and the activity of the fish was recorded for 5 minutes. The arena was virtually divided into center, middle, and thigmotaxis zones for data analysis. **B.** Schematic of the tank diving test. Fish were netted and placed into the novel tank (narrower and deeper than the home tank) and activity of the fish was recorded for 5 minutes. The tank was virtually divided into bottom, middle and top zones for data analysis.

Biology Open • Supplementary information
